# Does HIV infection have an impact upon dental implant osseointegration? A systematic review

**DOI:** 10.4317/medoral.20408

**Published:** 2015-02-07

**Authors:** Javier Ata-Ali, Fadi Ata-Ali, Nicolas Di-Benedetto, Leticia Bagán, José-Vicente Bagán

**Affiliations:** 1 DDS, MS, MPH, PhD. Public Dental Health Service. Arnau de Vilanova Hospital, Valencia, Spain; 2 DDS, MS. Valencia University Medical and Dental School; 3 MD. Infectious Diseases Unit, La Fe Hospital, Valencia Spain; 4 DDS. Valencia University Medical and Dental School; 5 MD, DDS, PhD, FDSRCSEd. Professor of Oral Medicine. Valencia University Medical and Dental School. Chairman Service of Stomatology and Maxillofacial Surgery, University General Hospital, Valencia, Spain

## Abstract

**Background:**

A systematic review is made to determine whether human immunodeficiency virus (HIV) infection has an impact upon dental implant osseointegration.

**Material and Methods:**

A PubMed (MEDLINE) literature search was made of articles published up until 14 April 2014. The systematic review was conducted based on the Preferred Reporting Items for Systematic Reviews and Meta-analysis (PRISMA). The quality of the studies included in the review was assessed using the Methodological Index for Nonrandomized Studies (MINORS) and levels of evidence (based on the University of Oxford’s Center for Evidence Based Medicine criteria).

**Results:**

The combinations of search terms resulted in a list of 132 titles. Nine studies finally met the inclusion criteria and were selected for inclusion in the systematic review. A total of 173 dental implants were placed in 80 patients (135 implants in 56 HIV-positive subjects and 38 implants in 24 HIV-negative patients), and a single loss of dental implant osseointegration was recorded in an HIV-positive patient.

**Conclusions:**

Our results suggest that dental implant placement in HIV-positive patients does not increase the dental implant failure rate. Prophylactic antibiotic treatment, the administration of highly active antiretroviral therapy, and control of the CD4+ T lymphocyte counts appear to be the main influencing factors in this respect. Given the few studies included in our systematic review, further prospective studies involving larger sample sizes and longer durations of follow-up are required in order to confirm the results obtained.

**Key words:** Dental implants, implant failure, HIV positive, systematic review, AIDS, HAART.

## Introduction

Human immunodeficiency virus (HIV) infection is a major public health problem. According to estimations of the United Nations, 34 million people throughout the world suffer from HIV/Acquired Immune Deficiency Syndrome (AIDS) (1).

The disease is characterized by progressive immune system failure that gives rise to the development of opportunistic infections and neoplasms. The virus invades CD4+ T lymphocytes, macrophages and dendritic cells, and the CD4+ T cell counts gradually decrease as a result of direct cytopathic action or cytotoxic CD8+T lymphocyte-mediated attack. Cellular immunity is affected once the lymphocyte count has dropped to below a critical point, and the patient becomes vulnerable to opportunistic infections. On the other hand, if HIV viral replication is not inhibited, the resulting immune activation increases the risk of cardiovascular events, tumors and kidney, liver and neurological disorders, among other problems (2-4). Following the introduction of highly active antiretroviral therapy (HAART) in 1996, the mortality rates associated with AIDS have decreased drastically, and enormous benefits have been obtained in terms of lessened patient morbidity and transmission of the infection. At present, thanks to the availability of increasingly effective and better tolerated antiretroviral treatments, the disease has been brought under control and the epidemic has been stabilized. This situation in turn has generated new challenges, for although HIV-infected individuals undergoing elective procedures and treatments have an adequate immune status, there are a number of factors that distinguish them from the general population, such as age (75% of the HIV-positive population is over 40 years of age), an increased prevalence of comorbidities, long-term complications of HAART, a greater need for medical care, the need for regular medication, and chronic inflammation (4-8). In this respect, it would be interesting to determine whether the dental implant osseointegration and success rates in HIV-infected individuals are the same as that observed in the general population. It should be taken into account that bone metabolic alterations are frequent in the context of HIV infection, due to a number of factors such as physical inactivity, depression, smoking, alcohol and opiate abuse, low testosterone levels, sub optimum calcium / vitamin D intake, and HAART (9).

A number of studies (10,11) have explored the association between different local and systemic factors and dental implant osseointegration. Buser *et al*. (11), on occasion of the second international team of oral implantology (ITI) consensus conference, proposed dividing the systemic risk factors affecting dental implant osseointegration into two groups: very high risk and significant risk. They concluded that immune depressed individuals, whether infected by HIV or subjected to immunosuppressive treatment (such as transplant patients), are at high risk.

Few data are found in the literature on dental procedures and their complications in HIV-infected patients, and there is limited experimental and clinical experience with dental implant placement in HIV infection. The present systematic review was therefore carried out to determine whether HIV infection has an impact upon dental implant osseointegration.

## Material and Methods

The Preferred Reporting Items for Systematic Reviews and Meta-analysis (PRISMA) statement was used in this study (12).

- PICO question

Does HIV infection have an impact upon dental implant osseointegration?

Search strategy for the identification of studies

The PubMed (MEDLINE) database of the United States National Library of Medicine was used for a literature search of articles published up until 14 April 2014. The following search terms were used in different combinations: “dental implant”, “AIDS”, “HIV”, “HIV-positive”, “HAART”, “HAART HIV”. Two examiners read the titles and abstracts of all studies, and no blinding was carried out regarding author names, journals or publication date. The search was completed with a review of the references of the selected articles in order to identify additional studies not found in the initial literature search.

In addition, a manual search (up until 14 April 2014) was made of the following journals: Clinical Implant Dentistry and Related Research, Clinical Oral Investigations, Clinical Oral Implants Research, Implant Dentistry, International Journal of Oral and Maxillofacial Implants, Journal of Clinical Periodontology, Journal of Oral Implantology, Journal of Oral and Maxillofacial Surgery, Journal of Periodontology, Medicina Oral, Patologıa Oral y Cirugıa Bucal, and Oral Surgery and Oral Medicine, Oral Pathology, Oral Radiology, and Endodontology.

- Study selection criteria

Before starting the study, a series of inclusion and exclusion criteria were established. Chosen full-text articles were assessed for the following inclusion criteria: (a) Studies including HIV-infected patients receiving at least one dental implant; (b) Prospective and retrospective studies, case series and case reports. In vitro or animal studies were excluded. Authors were contacted for clarification of missing information when necessary. No restrictions were placed on the year or language of publication. All articles selected from the electronic and manual searches were independently assessed by the first and second authors of the present study, according to the established inclusion criteria. Any disagreements between the reviewing authors were resolved by consensus, or by consulting the last signing author of the study.

- Quality assessment

Two authors independently evaluated the quality of the studies included in the systematic review using the Methodological Index for Nonrandomized Studies (MINORS) (13). The MINORS scale includes the following points: (a) a clearly stated aim; (b) inclusion of consecutive patients; (c) prospective collection of data; (d) appropriate endpoints; (e) unbiased assessment; (f) a follow-up period; (g) losses to follow-up of < 5%; and (h) prospective calculation of the study size for non-comparative studies ( Table 1), and additional criteria in the case of comparative studies; (i) an adequate control group; (j) contemporary groups; (k) baseline equivalence of groups; and (l) adequate statistical analyses ( Table 2). The items on the MINORS scale are scored as 0 (not reported), 1 (reported but inadequate) or 2 (reported and adequate). The ideal global score is 16 for non-comparative studies and 24 for comparative studies. Furthermore, we defined study quality as poor (< 5), fair (6-10) or good (> 11). Quality was also assessed according to the levels of evidence (based on the University of Oxford’s Center for Evidence Based Medicine criteria) (23) ( Table 1 and  Table 2).

**Table 1:**
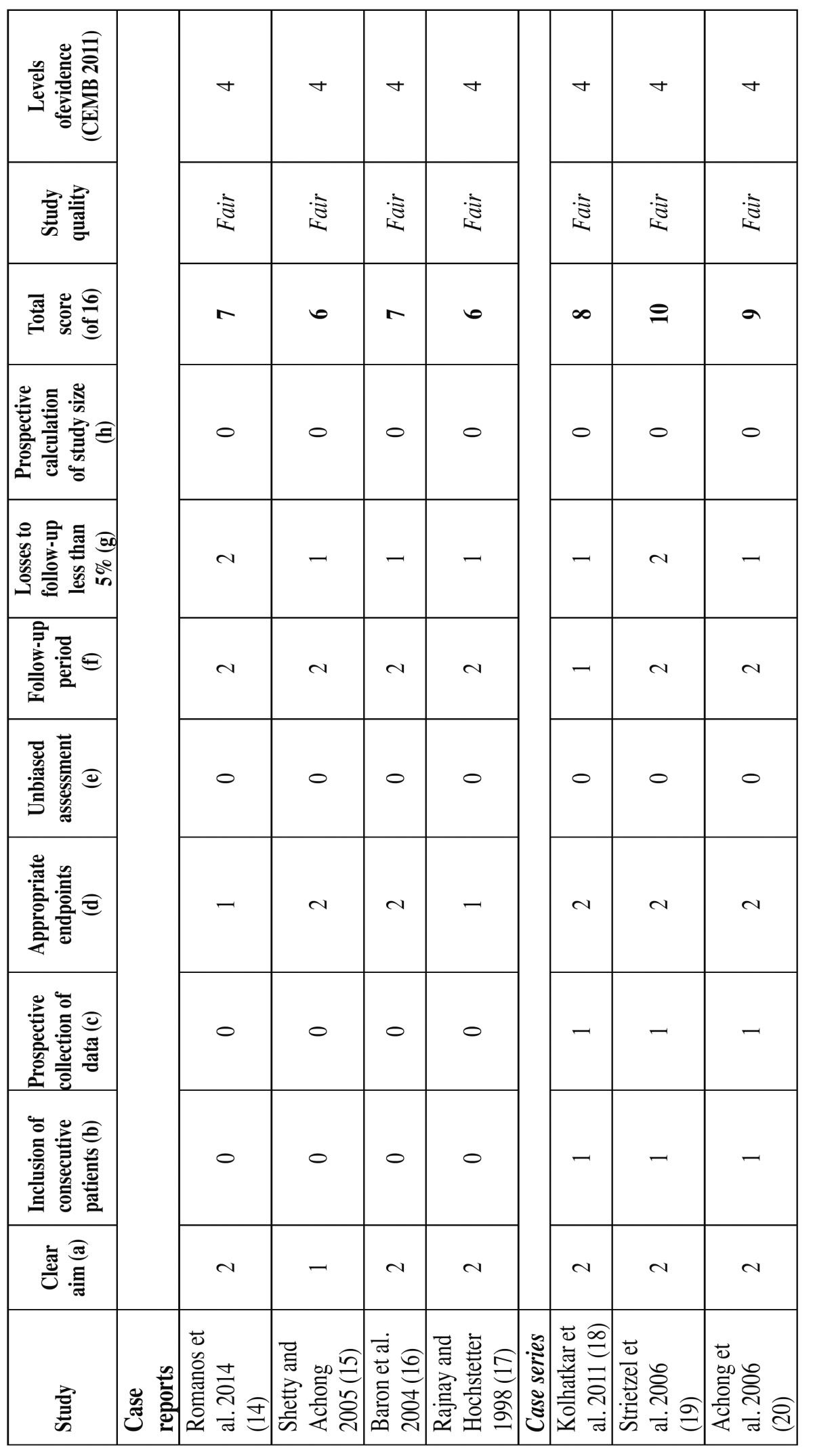
Quality assessment scores of case series and case reports using the 8-point MINORS scale and levels of evidence (CEMB 2011).

**Table 2:**
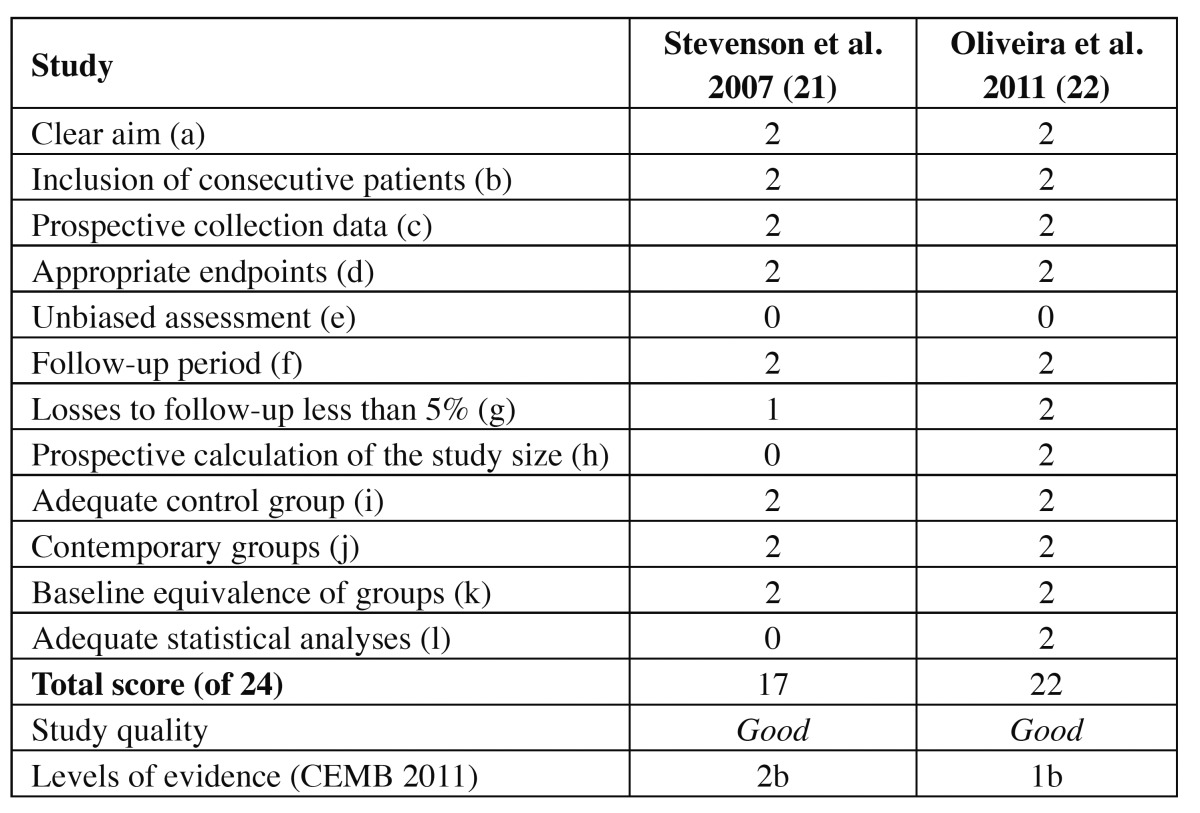
Quality assessment scores of prospective studies using the 12-point MINORS scale and levels of evidence (CEMB 2011).

## Results

- Study selection

**Figure 1:**
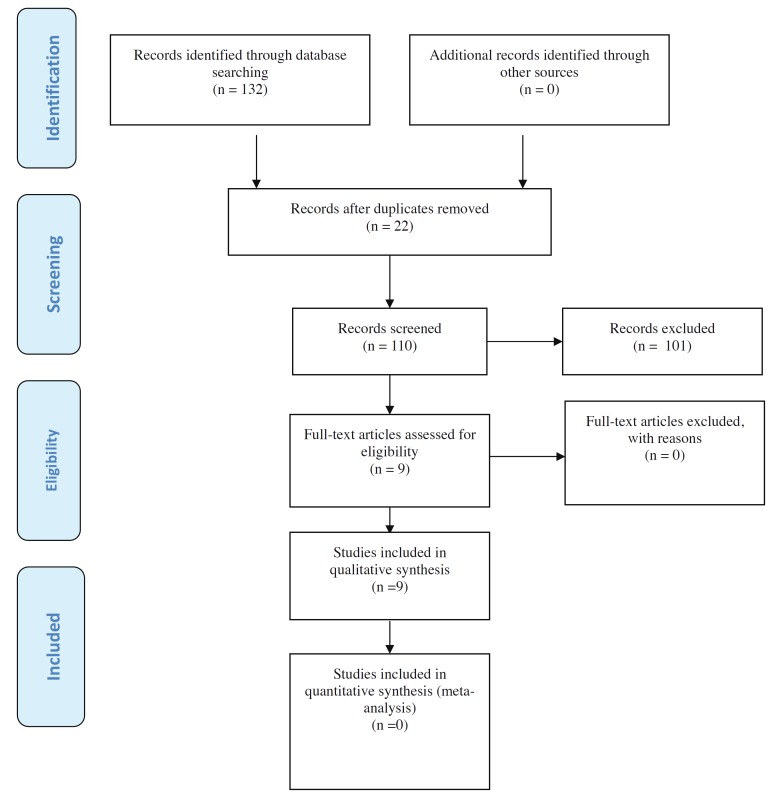
Prisma® flow diagram of the search processes and results.

The combinations of search terms resulted in a list of 132 titles. Of these, 22 were found to be duplicated; as a result, 110 references were reviewed. Subsequently, 101 papers were excluded on the basis of the evaluation of the title and abstract, thus leaving 9 articles for eligibility assessment. Nine publications finally met the inclusion criteria and were thus selected for inclusion in the systematic review (Fig. 1).

- Assessment of study quality

Two reviewers (JAA and FAA) independently and in duplicate evaluated the quality of the included studies as part of the data extraction process. Any disagreements were resolved by consensus or by consulting the last signing author of the present study. The mean score for all case series was 9, with a range of 8-10. The mean score for all case reports was 6.5, with a range of 6-7. This suggests fair quality of the included non-comparative studies. The mean score for all prospective studies was 19.5, with a range of 17-22, which suggests good quality of the included comparative studies. In the level of evidence assessment, 7 studies (14-20) ranked as level 4, one study (21) corresponded to level 2b, and one study (22) was ranked as level 1b.

- Description of the studies

Of the 9 studies included in the systematic review, four were case reports (14-17), three were case series (18-20), and two were prospective studies (21,22). The demographic data (patient age and sex) and information referred to the dental implants (number and type, failed implants, location and follow-up) of the publications are summarized in  Table 3. The laboratory test data (CD4+ T lymphocyte count and viral load) and information referred to antiretroviral therapy and preventive treatment in turn are summarized in  Table 4. In the present systematic review, a total of 173 dental implants were placed in 80 patients (135 implants in 56 HIV-positive subjects and 38 implants in 24 HIV-negative patients belonging to the control groups of the prospective studies) (21,22). A single loss of dental implant osseointegration was recorded in an HIV-positive patient.

**Table 3:**
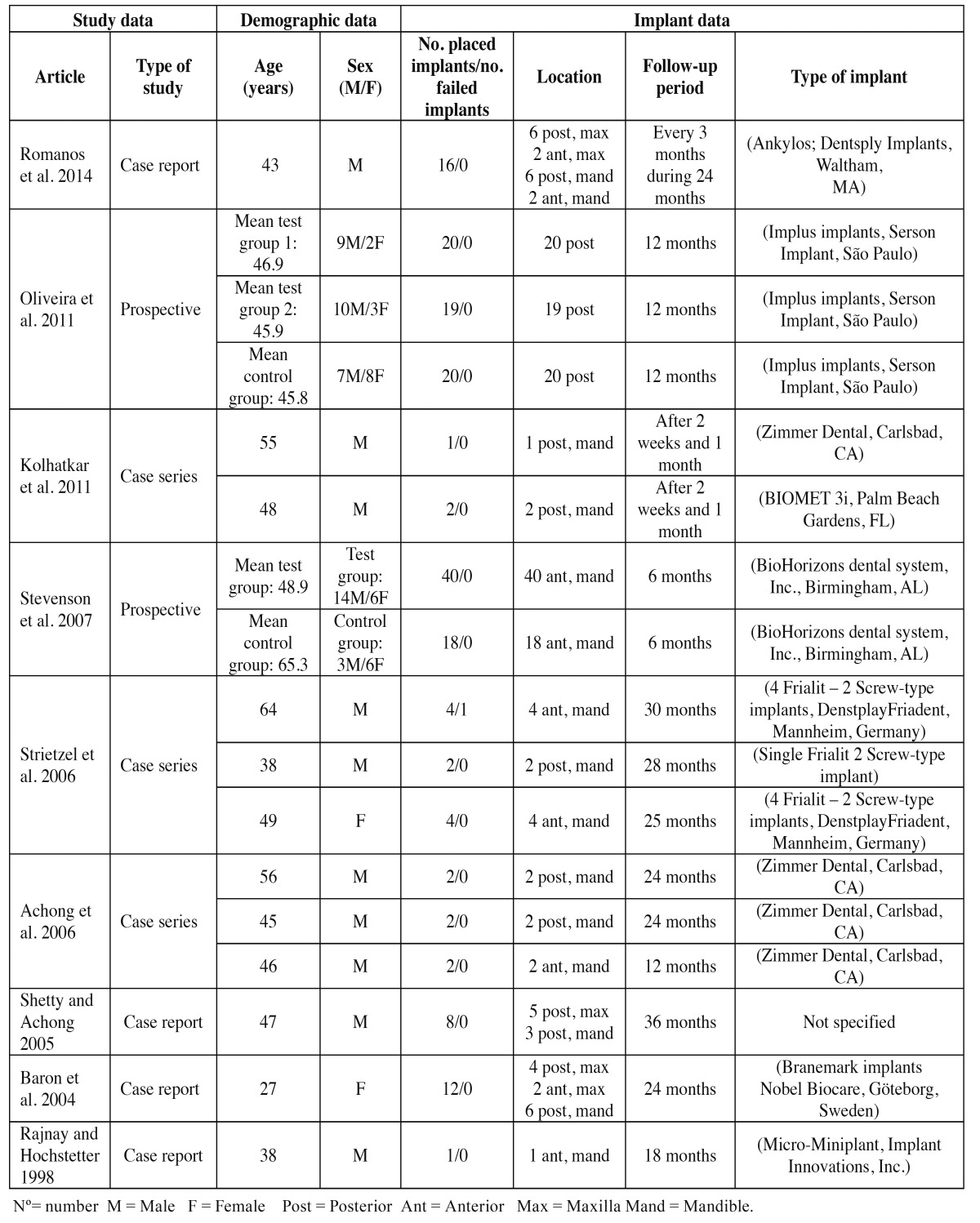
Demographic and dental implant data of the studies included in the systematic review.

**Table 4:**
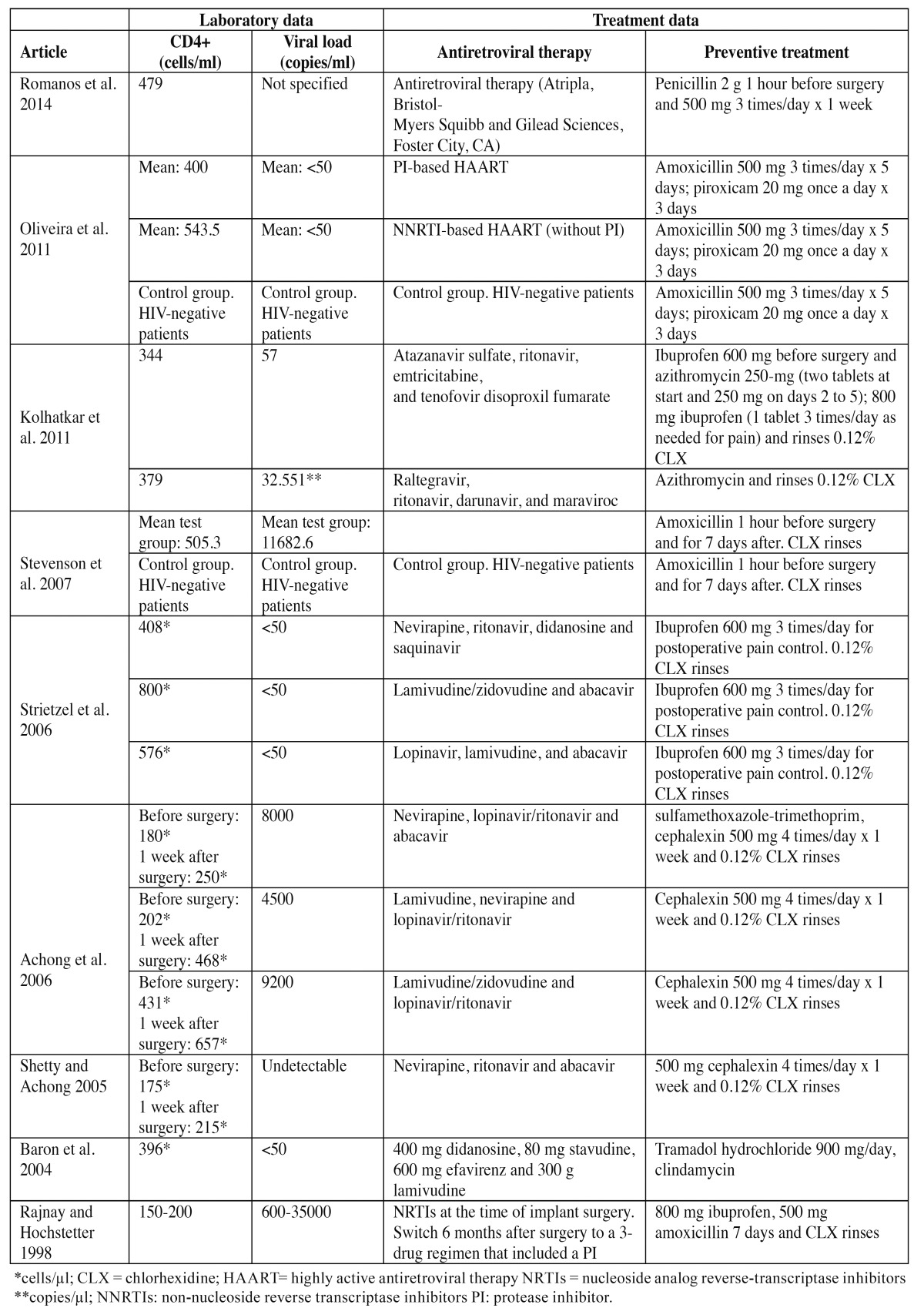
Laboratory test and treatment data of the studies included in the systematic review.

## Discussion

The present systematic review has examined the scientific evidence with a view to determining the possible impact of HIV infection upon dental implant osseointegration. We analyzed a total of 9 studies in which 173 implants were placed, and only one implant osseointegration failure was recorded, corresponding to an HIV-infected individual.

Since the introduction of HAART in 1996, survival among HIV-infected individuals has greatly improved, and new challenges have appeared. In this respect, dental implant placement in HIV-infected patients is increasingly common, though the impact of HIV infection upon the success of implant osseointegration has not been fully established. The studies published to date (24,25) have not found major surgery to have a negative impact upon patients with HIV infection. Studies conducted in the early post-HAART era (24,25) have shown the evolution of CD4+ T cell counts in surgical patients to be similar to that seen in individuals not subjected to surgery. However, although surgery does not influence the evolution of HIV infection, the latter might influence the outcome of surgery. In this regard, conflicting results have been obtained by studies that have examined the effect of HIV infection upon the success and tolerance of surgery (26-34). Some articles, mostly published in the pre- or early post-HAART era (26-31), have described more postoperative complications in HIV-infected individuals than in the general population. The most frequently reported problems have been bacterial infections, with CD4+ T lymphocyte count and viral burden as underlying risk factors. However, some recent studies (32-34) on the efficacy and tolerability of different surgical procedures in HIV-infected patients have recorded data similar to those found in the general population.

The natural history of HIV infection has changed drastically since the introduction of HAART. In effect, the availability of effective and well tolerated antiretroviral treatments has led to a notorious increase in patient survival, with a lesser incidence of AIDS-defining diseases. Furthermore, the causes of death have changed, and the associated chronic disorders have grown in importance (8).

In the HIV-positive population, bone metabolic disorders have become common as a result of the improvements in life expectancy. The most frequently reported bone disorders in these subjects are related to bone demineralization, such as osteoporosis and osteopenia (9,35,36). A study in HIV-infected patients has recorded pre valences of osteopenia and osteoporosis of 48% and 23%, respectively (37). Osteoporosis is characterized by a decrease in bone density and mineral content in peripheral bone, associated to increased maxillary resorption and atrophy. However, there is no associated increase in dental implant loss (38). A study involving the placement of 82 dental implants in 39 patients (including 39 implants in 19 osteoporotic patients) recorded no statistically significant differences between the patients with osteoporosis and those without – the dental implant success rate being 98.8% (39). In a retrospective study of 70 dental implants in osteoporotic patients, the implant success rate was found to be 97% after over three years of follow-up (40).

A number of factors might contribute to the high prevalence of such bone metabolic disorders in HIV-infected patients. In this respect, it is possible that no single factor can explain the development of these disorders; rather, the underlying cause may be a combination of factors that are characteristically more prevalent in such individuals, such as low body weight, malnutrition, malabsorption, sub optimum calcium / vitamin D intake, physical inactivity, low testosterone levels, smoking, alcohol and other substance abuse, HIV disease itself, and HAART (9). The proposed mechanisms whereby the virus could contribute to the loss of bone mineral density are related to osteoclast stimulation and diminished bone production on the part of the osteoblasts. The stimulation of osteoclast activity would be a response to the increased production of pro inflammatory cytokines secondary to chronic T cell activation, while diminished bone production on the part of the osteoblasts would be a consequence of increased apoptosis among these cells (41-43). Certain drugs, such as the bisphosphonates, significantly reduce bone turnover. It is therefore not surprising that a patient taking bisphosphonates may have a problem with dental implant integration (44), with the associated risk of osteonecrosis of the jaws (45). However, several studies (46-48) have shown the dental implant failure rate in patients who receive these drugs to be similar to that seen in patients who do not receive such treatments.

In a study of 40 dental implants placed in 20 HIV-infected patients, no implant osseointegration failures were recorded after 6 months of follow-up (21). Likewise, in another study of 39 dental implants placed in 24 HIV-infected patients, no implant osseointegration failures were recorded after one year of follow-up (22). The above findings have been corroborated by several studies (14-18,20) in HIV-infected individuals in which no dental implant osseointegration failures were observed. Of the 173 dental implants included in our systematic review, only one osseointegration failure was documented, corresponding to an implant placed in the lower anterior sector in a woman, after 30 months of follow-up (19).

It is logical to assume that antibiotic use is indicated in HIV-negative patients, as has been demonstrated in a meta-analysis in which antibiotic use significantly lowered the implant failure rate (p = 0.003), with an odds ratio of 0.331 - thus implying that antibiotic treatment reduced the odds of implant failure by 66.9% (49). It therefore can be postulated that antibiotics should also be prescribed in HIV-positive individuals in order both to reduce implant failure and to minimize the risk of postoperative infections.

- Study limitations

The main limitation of this systematic review is the small number of articles that were available for evaluation. Our aim was to conduct a meta-analysis to determine whether human immunodeficiency virus (HIV) infection has an impact upon dental implant osseointegration. This was not possible mainly because of the heterogeneity of the studies in relation to the types of implants used, and the differences in antiretroviral therapy, preventive treatment (antibiotics and analgesics), and follow-up periods. Another limitation is the fact that our systematic review included publications with a lower level of evidence than randomized controlled trials (RCTs). The absence of randomized controlled trials therefore means that our review is based on rather limited evidence.

## Conclusions

Based on the results of our systematic review of the literature, it seems that the prognosis of dental implant placement in HIV-infected patients is good and similar to that seen in HIV-negative individuals. This is particularly manifest in the presence of HAART, controlled CD4+ T lymphocyte counts, and the administration of prophylactic antibiotic therapy. However, further prospective studies involving larger sample sizes and longer durations of follow-up are required in order to confirm the results obtained.
